# The Effect of Postoperative Sepsis on 1-Year Mortality and Cancer Recurrence Following Transhiatal Esophagectomy for Esophageal–Gastric Junction Adenocarcinomas: A Retrospective Observational Study

**DOI:** 10.3390/cancers17010109

**Published:** 2025-01-01

**Authors:** Marion Faucher, Samuel Dahan, Bastien Morel, Jean Manuel de Guibert, Laurent Chow-Chine, Frédéric Gonzalez, Magali Bisbal, Luca Servan, Antoine Sannini, Marie Tezier, Maxime Tourret, Sylvie Cambon, Camille Pouliquen, Damien Mallet, Lam Nguyen Duong, Florence Ettori, Djamel Mokart

**Affiliations:** Department of Anesthesiology and Critical Care, Paoli-Calmettes Institute, 13009 Marseille, France; faucherm@ipc.unicancer.fr (M.F.); dahansamuel1@gmail.com (S.D.); morelb@ipc.unicancer.fr (B.M.); deguibertj@ipc.unicancer.fr (J.M.d.G.); chowchinel@ipc.unicancer.fr (L.C.-C.); gonzalezf@ipc.unicancer.fr (F.G.); bisbalm@ipc.unicancer.fr (M.B.); servanl@ipc.unicancer.fr (L.S.); sanninia@ipc.unicancer.fr (A.S.); tezierm@ipc.unicancer.fr (M.T.); tourretm@ipc.unicancer.fr (M.T.); cambons@ipc.unicancer.fr (S.C.); pouliquenc@ipc.unicancer.fr (C.P.); malletd@ipc.unicancer.fr (D.M.); nguyenduongl@ipc.unicancer.fr (L.N.D.); ettorif@ipc.unicancer.fr (F.E.)

**Keywords:** esophageal cancer, surgery, sepsis, mortality, cancer recurrence

## Abstract

Introduction: Transhiatal esophagectomy (THE) is used for specific gastroesophageal junction adenocarcinomas. THE is a high-risk surgical procedure. We aimed to assess the impact of postoperative sepsis (sepsis or septic shock) on the 1-year mortality after THE and to determine the risk factors associated with these outcomes. Secondly, we aimed to assess the impact of postoperative sepsis and other risk factors on 1-year cancer recurrence. Method: A retrospective, observational study was undertaken at the Paoli-Calmettes Institute, Marseille, from January 2012 to March 2022. Results: Of 118 patients, 24.6% (n = 29) presented with postoperative sepsis. Their 1-year mortality was 11% (n = 13), and their 1-year cancer recurrence was 23.7% (n = 28). In the multivariate analysis, independent factors for 1-year mortality were the following: postoperative sepsis (OR: 7.22 (1.11–47); *p* = 0.038), number of lymph nodes removed (OR: 0. 78 (0.64–0.95); *p* = 0.011), recurrence at one year (OR: 9.22 (1.66–51.1); *p* = 0.011), mediastinitis (OR: 17.7 (1.43–220); *p* = 0.025) and intraoperative driving pressure (OR: 1.77 (1.17–2.68); *p* = 0.015). For postoperative sepsis, independent factors were low-dose vasopressors (OR: 0.26; 95% CI: 0.07–0.95; *p* = 0.049), a cervical abscess (OR: 5.33; 95% CI: 1.5–18.9; *p* = 0.01), bacterial pneumonia (OR: 11.1; 95% CI: 2.99–41.0; *p* < 0.001) and a high SOFA score on day 1 (OR: 2.65; 95% CI: 1.36–5.19; *p* = 0.04). For 1-year cancer recurrence, independent factors were the number of lymph nodes removed (sHR: 0.87; 95% CI: 0.79–0.96; *p* = 0.005), pTNM stages of III or IV (sHR: 8.29; 95% CI: 2.71–25.32; *p* < 0.001) and postoperative sepsis (sHR: 6.54; 95% CI: 1.70–25.13; *p* = 0.005). Conclusions: Our study indicates that after THE, postoperative sepsis influences survival and cancer recurrence. We identified the associated risk factors, suggesting an early diagnosis might decrease mortality and recurrence.

## Simple Summary

This study investigated the effects of postoperative sepsis on the survival and cancer recurrence rates of patients who undergo a specific type of surgery called transhiatal esophagectomy, often used to treat cancer of the gastroesophageal junction. The surgery is high-risk, and complications such as sepsis can have serious consequences. The authors aimed to understand how sepsis impacts one-year survival and cancer recurrence rates and identify factors that increase the risk of these outcomes. The findings suggest that sepsis significantly affects both survival and cancer recurrence, with factors such as the number of lymph nodes removed and the presence of certain complications influencing the outcomes. By identifying these risk factors, this study highlights the importance of early diagnosis and treatment to improve patient outcomes after surgery.

## 1. Introduction

The incidence of adenocarcinomas (ADKs) at the esophageal–gastric junction (EGJ) is increasing globally, particularly among esophageal cancers [1,2]. The overall 5-year survival rate for these tumors is approximately 13%, improving to 50% for localized forms [3]. For localized EGJ adenocarcinomas classified as Siewert types 1 and 2, esophagectomy is the recommended treatment [4]. Traditionally, the gold standard has been transthoracic esophagectomy (TTE), which involves abdominal, thoracic and sometimes cervical access [5,6]. However, TTE is associated with significant postoperative morbidity, with up to 57% of patients suffering from postoperative complications [7]. Transhiatal esophagectomy (THE) serves as an alternative, employing cervical and abdominal approaches, and demonstrates comparable oncological outcomes to those of TTE [8,9,10,11,12]. Postoperatively, THE may offer advantages, including reduced mortality [8,13], fewer septic and respiratory complications [7,8] and shorter hospital stays [7,8]. THE may be conducted using a wide bi-subcostal laparotomy or a minimally invasive laparoscopic approach. Nonetheless, there is a lack of randomized studies directly comparing these techniques, and robust evidence favoring one over the other is currently absent [12]. THE remains a high-risk procedure, with septic complications reported in up to 50% [14] of cases and a 30-day postoperative mortality rate between 2.8% and 7.2% also reported [8]. Studies indicate a strong correlation between postoperative complications following major surgery and 1-year mortality rates [15,16]. Additionally, evidence indicates a potential association between cancer recurrence and notable complications, particularly in cases involving postoperative sepsis [17,18,19,20,21,22]. Hence, the early identification and management of risk factors for postoperative sepsis are crucial for patients undergoing major oncological surgery [23].

First, we aimed to assess the impact of postoperative sepsis on 1-year mortality after THE and to determine the risk factors associated with these outcomes. Secondly, we aimed to assess the impact of postoperative sepsis and other risk factors on 1-year recurrence.

## 2. Materials and Methods

### 2.1. Study Design

This retrospective observational study was conducted at the Paoli-Calmettes Institute, a cancer referral center in Marseille, France, with ethical approval (DAR-OESO-IPC 2022-31) provided by the institutional review board, which waived the need for informed consent on 26 April 2022. This study included all adult patients who underwent THE for adenocarcinoma of the EGJ from January 2012 to March 2022, all of whom received chemotherapy or chemoradiotherapy. Patients with intraoperative findings of advanced disease that led to cancelled surgery, those undergoing alternative surgical procedures and those who experienced severe complications on the day of surgery (Day 0) were excluded from this study. Patients scheduled for THE were admitted to the intermediate care or intensive care unit (IMC/ICU) for standard monitoring and postoperative care for a minimum of three days before their transfer to the surgical ward, except in the event of complications.

### 2.2. Definitions

One-year mortality was defined as death from any cause within one-year post-surgery. The Clavien–Dindo classification assessed postoperative complications per the international standards for esophagectomy [24,25]. Severe complications were classified as grade IIIb or higher [26]. Postoperative acute respiratory failure (ARF) was identified clinically through tachypnea, the use of accessory respiratory muscles, an arterial oxygen saturation below 90%, pulmonary infiltrates, or the need for high-concentration oxygen, invasive mechanical ventilation (IMV), or non-invasive ventilation (NIV). Postoperative sepsis (sepsis or septic shock) was defined according to the Sepsis-3 criteria [27]. Shock was generally defined as persistent hypotension necessitating vasopressor support for a MAP ≥ 65 mm Hg. Acute kidney injury (AKI) was defined according to the KDIGO criteria [28], while malnutrition was assessed based on the French diagnostic criteria [29]. Other postoperative organ failures were evaluated using the SOFA score on postoperative days 1 and 3.

### 2.3. Intraoperative Care

A standardized protocol for general anesthesia was implemented for all patients. The preferred surgical approach was laparoscopy, with a transition to bi-subcostal laparotomy as required. Further details on the anesthesia, surgical management, and pathology results are available in Supplementary Files S1–S3 [30,31].

### 2.4. Postoperative Care

While our institute has adopted the ERAS protocols since 2016 for high-risk surgeries, there is no specific protocol for esophageal surgery [32]. However, over the decade, practices shifted towards early mobilization, quicker oral nutrition, and decreased use of intraoperative jejunostomy [32]. In our IMC/ICU, we systematically treat postoperative sepsis and septic shock with a local protocol that complies with the fundamental elements of recent guidelines [33].

### 2.5. Data Collection

The key data for evaluation are presented in Table 1, Table 2 and Table 3, including age, BMI, malnutrition, smoking status, cardiovascular comorbidities, preoperative radiotherapy or chemotherapy, Charlson Comorbidity Index, American Society of Anesthesiologists (ASA) physical status, Simplified Acute Physiology Score (SAPS II) and Sequential Organ Failure Assessment (SOFA) score, all recorded during the perioperative period. Cancer type, diagnosis date and metastatic nature were also documented. Details regarding the surgical procedure, anesthetic protocol and intraoperative complications were recorded, along with the administration of intraoperative vasopressors and ventilatory parameters (tidal volume, plateau pressure, positive end-expiratory pressure and FiO_2_). The driving pressure during intraoperative ventilation was averaged per patient and treated as a continuous variable [34]. Severe postoperative complications, including AKI, ARF, shock, postoperative sepsis, reoperation or death, were recorded within 30 days of surgery and classified according to the Clavien–Dindo classification. Interventions during the IMC/ICU stay, and hospital lengths of stay were collected. Long-term outcomes were assessed with a follow-up period of one year, while postoperative complications were documented within 30 days postoperatively.

## 3. Outcomes

This study evaluated two primary outcomes: all-cause mortality within one-year post-surgery and the incidence of postoperative sepsis within 30 days of surgery. The secondary outcome was cancer recurrence within one year of surgery. Cancer recurrence was defined as an inclusive state that included recurrence without progression or progression. The secondary endpoint was disease-free survival (DFS). DFS was defined as the interval between the date of surgery and the date of cancer recurrence or last follow-up.

### 3.1. Follow-Up

Patients were monitored for one-year post-surgery using the hospital’s electronic system, which records all procedures, visits, laboratory tests, vital signs and other relevant data, complete with dates and unique identifiers. The Paoli-Calmettes Institute adheres to a policy of regular follow-ups, scheduling at least one visit every three months following major oncological surgery. In cases of patients lost to follow-up, the INSEE database was employed to ascertain their deceased status (https://arbre.app/insee, access on 10 September 2023).

### 3.2. Statistical Analysis

The data are presented as percentages for qualitative variables and as medians (interquartile ranges) for quantitative variables. Comparisons were performed between patients who died within one-year post-surgery and those who survived. The Mann–Whitney test was used for continuous variables, while Chi-square or Fisher tests were used for categorical variables, with statistical significance defined as *p* < 0.05. Logistic regression analyses identified independent variables associated with 1-year mortality, providing estimated odds ratios (ORs) and 95% confidence intervals (CIs). Significant variables, those of borderline significance (*p* < 0.1) and clinically relevant variables were included in the multivariate regression model. Then, they were entered into the multivariable model with variable selection based on the Akaike information criterion (AIC). In the second part, we analyzed factors associated with the occurrence of postoperative sepsis within 30 days post-op using the same statistical method as that described above, focusing specifically on the incidence of these complications. Finally, we analyzed factors associated with 1-year cancer recurrence using a competing risk analysis, as described by Fine and Gray. This method takes into account the risk of death before recurrence. The univariate analyses focused on factors with a *p*-value < 0.1 and those deemed relevant in the literature for a subsequent multivariate analysis via Fine and Gray’s proportional hazard models with AIC selection. It was planned to test the influence of postoperative sepsis a priori in the multivariate analysis, even though this variable was not selected in the univariate analysis. The results are presented as sub-distribution hazard ratios (sHRs) with 95% CIs to assess the association between the covariates and cancer recurrence. A Sankey network diagram was used to visualize the interactions between the significant clinical features and the postoperative sepsis group. The probability of 1-year survival was represented using a Kaplan–Meier curve, and differences among groups was evaluated using the log-rank test. Adjusted cumulative incidence curves were used to describe the cumulative incidence of 1-year cancer recurrence according to postoperative sepsis. All tests were two-sided, and *p* values < 0.05 were considered statistically significant. The analyses were performed using R software, version 4.3.3 (R Foundation for Statistical Computing, Vienna, AT).

## 4. Results

**Figure 1:**
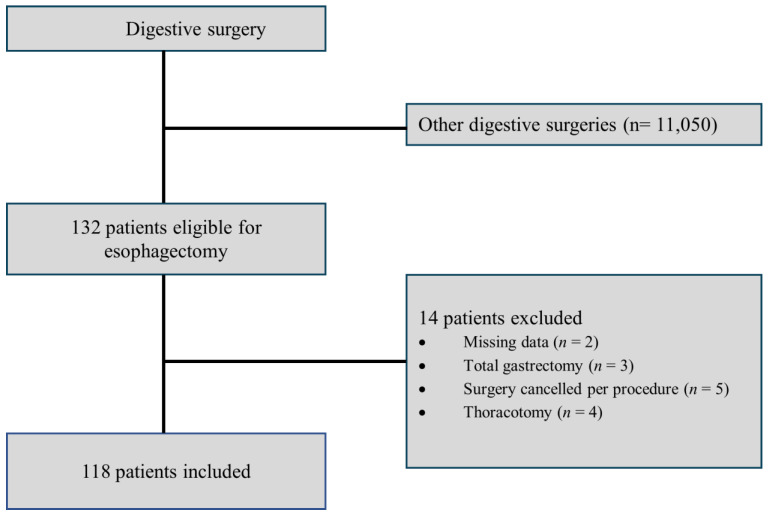
Flow chart—the study population between January 2012 and March 2022.

Between January 2012 and March 2022, 45,546 patients underwent surgery at the Institut Paoli-Calmettes. Of these, 11,082 had digestive surgery, with 132 being eligible for THE. Following the exclusion of 14 patients, 118 underwent THE surgery and were included in the analysis (Figure 1).

### 4.1. The Pre- and Intraoperative Periods

The cohort had a median age of 64 years (57–70) at the time of surgery, with 85.6% (n = 101) of them being men. Their BMI was 24.7 (22.6–26.6), and 27 patients (22.9) presented with an ASA score > 2. Arterial hypertension was observed in 33.4% (n = 40) of the patients, while chronic lung disease was present in 14.4% (n = 17). A history of smoking was reported in 68.6% (n = 81) of them, and 6.8% (n = 8) had risky alcohol consumption. Malnutrition affected 39.8% (n = 47). Preoperative chemotherapy was administered to 88.9% (n = 104) and radiotherapy to 44% (n = 51). Laparoscopy was performed in 55.9% (n = 66) of cases, while 44.1% (n = 52) underwent extended laparotomy. Low-dose vasopressors were given to 72.9% (n = 86) of cases, with standard-dose vasopressors used in 3.4% (n = 4). The intraoperative fluid volume was 4.26 L (3.38–5.18), with a tidal volume of 6.8 mL/kg (6.3–7.1). PEEP measured 5.9 cmH_2_O (4.9–6.4), and driving pressure was 8.9 cmH_2_O (7.4–10). Abdominal drains were placed in 99.2% (n = 117) of cases, cervical drainage in 89.8% (n = 106) of cases and thoracic drainage in one case. An epidural catheter was used in 70.3% (n = 83), while 16.1% (n = 19) received spinal anesthesia. The surgical duration was 5.55 h (4.86–6.49).

### 4.2. The Postoperative Period

A severe complication occurred in 42.4% (n = 50) of the patients. Among these, 13.5% (n = 16) experienced shock, comprising 8 with septic shock, 4 with hemorrhagic shock and 4 with other types. ARF was reported in 28.8% (n = 34) of the patients and postoperative sepsis in 26.4% (n = 29); see Table 4. Postoperative sepsis occurred 3 (2–5) days after surgery and was never diagnosed before day 2. The overall mortality rate within the year was 11% (n = 13), with 7 patients experiencing recurrence at the time of death. A total of 28 patients (23.7%) experienced recurrence during the year, and only 1 was lost to follow-up, which did not interfere with a diagnosis of recurrence. Post-admission, the SAPS II score was 17 (12–22). Complications included abdominal abscesses (18.6%, n = 22), mediastinitis (5.9%, n = 7), anastomotic fistulae (28%, n = 33) and cervical abscesses (28.8%, n = 34). Among the patients diagnosed with a cervical abscess, its etiology was identified as an inadequately drained anastomotic leak in 26 cases (77%), while in the remaining 8 cases (23%), the cause was undetermined (*p* < 0.001); however, it was not possible to definitively exclude that an inadequately drained anastomotic leak contributed to their status. Pleural effusion was noted in 50% (n = 60) of cases, and pneumothoraxes occurred in 15.3% (n = 18). Postoperative drainage was necessary in 17.8% (n = 21) of the patients. Pericardial effusion was observed in 5% of them (n = 6). The interventions comprised invasive mechanical ventilation (IMV) in 7.6% (n = 9), non-invasive mechanical ventilation (NIV) in 18.6% (n = 22) and a high-flow nasal cannula (HFNC) in 17.8% (n = 21). Atrial fibrillation was documented in 21.2% (n = 25) and dysphonia in 10.2% (n = 12) of the patients. A surgical reoperation was required for 22% (n = 26) of the patients. Their 30-day and 90-day mortality rates were 1.7% (n = 2) and 2.5% (n = 3), respectively. Further detailed results are detailed in Table 1 and Table 2.

### 4.3. Risk Factors for 1-Year Mortality

**Figure 2:**
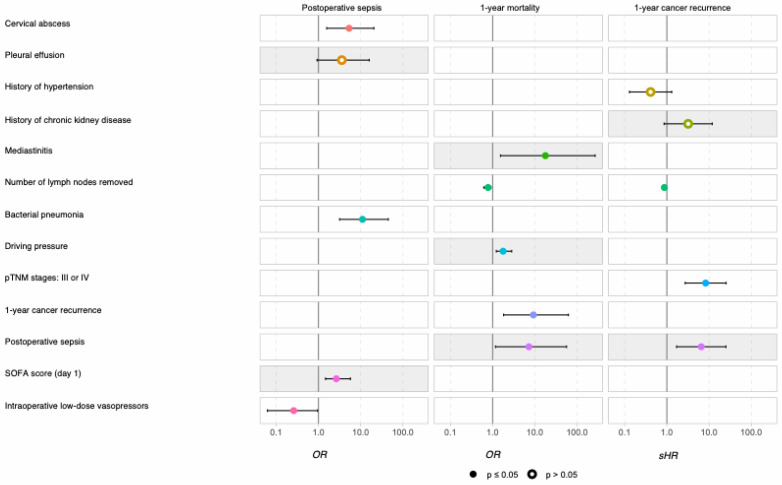
Multivariate analyses for factors independently associated with postoperative sepsis, 1-year mortality and 1-year cancer recurrence.

**Figure 3:**
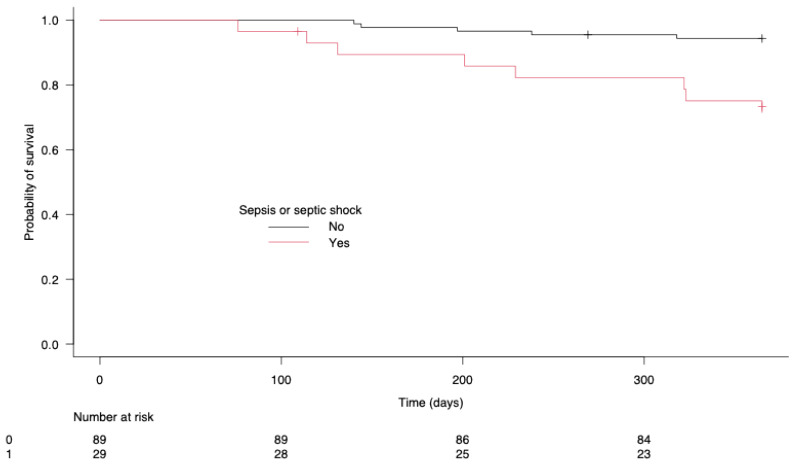
One-year survival of patients according to postoperative sepsis.

The univariate analysis (Table 1) showed significant associations between 1-year mortality and several variables: driving pressure (*p* = 0.041), mediastinitis (*p* = 0.033), pericardial effusion (*p* = 0.014), acute respiratory distress syndrome (ARDS) (*p* = 0.001), IMV (*p* = 0.005), postoperative fever (*p* = 0.011), AKI (*p* = 0.021), postoperative sepsis (*p* = 0.003), the number of lymph nodes removed (*p* = 0.011) and cancer recurrence at 1 year (*p* = 0.050). The multivariate analysis (Figure 2) identified five independent predictors of 1-year mortality: postoperative sepsis (OR: 7.22 (1.11–47); *p* = 0.038), the number of lymph nodes removed (OR: 0. 78 (0.64–0.95); *p* = 0.011), recurrence at one year (OR: 9.22 (1.66–51.1); *p* = 0.011), mediastinitis (OR: 17.7 (1.43–220); *p* = 0.025) and intraoperative driving pressure (OR: 1.77 (1.17–2.68); *p* = 0.015); see Figure 2. Figure 3 shows the 1-year survival rates according to postoperative sepsis (*p* = 0.007).

### 4.4. Risk Factors for Sepsis or Septic Shock

**Figure 4:**
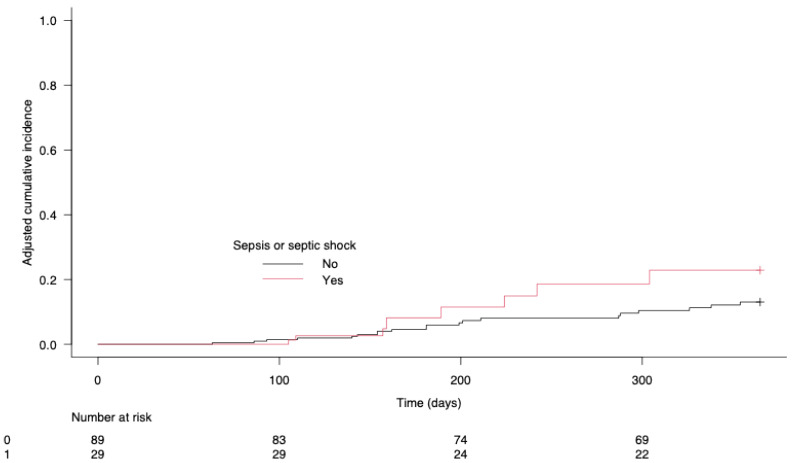
Adjusted cumulative incidence of 1-year cancer recurrence according to postoperative sepsis.

According to the results of the univariate analysis presented in Table 2, several variables were found to be significantly associated with the occurrence of postoperative sepsis. These variables included the use of low-dose vasopressors (*p* < 0.001), SOFA score on day 1 (*p* = 0.004), the presence of an anastomotic fistula (*p* < 0.001), pleural effusion (*p* < 0. 001), pericardial effusion (*p* = 0.003), bacterial pneumonia (*p* < 0.001), a cervical abscess (*p* = 0.004), atelectasis (*p* < 0.001), postoperative fever (*p* ≤ 0.001) and reoperation (*p* < 0.001). The multivariate analysis presented in Figure 4 identified four factors as independent predictors of postoperative sepsis: the use of intraoperative low-dose vasopressors (OR: 0.26; 95% CI: 0.07–0. 95; *p* = 0. 049), a cervical abscess (OR: 5.33; 95% CI: 1.5–18.9; *p* = 0.01), bacterial pneumonia (OR: 11.1; 95% CI: 2.99–41.0; *p* < 0.001) and a high SOFA score on day 1 (OR: 2.65; 95% CI: 1.36–5.19; *p* = 0.04); see Figure 2.

### 4.5. Risk Factors for 1-Year Cancer Recurrence

**Figure 5:**
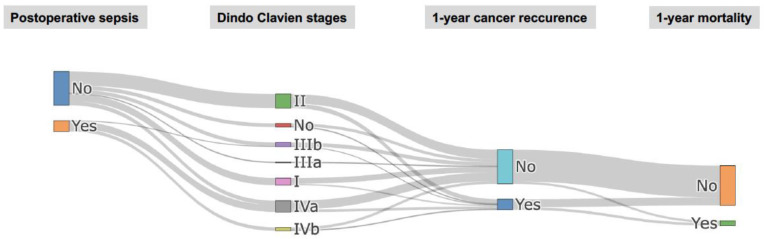
Relationship between different clinical statuses and postoperative sepsis. A Sankey diagram representing the relationship between postoperative sepsis, Clavien–Dindo stages, cancer recurrence and mortality, with the link widths representing the absolute number of patients.

The univariate analysis (Table 3) showed several factors significantly associated with cancer recurrence at one year: BMI (*p* = 0.048), postoperative platelet transfusion (*p* = 0.031), fresh frozen plasma transfusion (*p* = 0.01), pTNM stages 3 or 4 (*p* < 0.001) and incomplete tumor resection (*p* = 0.001). The multivariate analysis identified independent associations with recurrence, including the number of lymph nodes removed (sHR: 0.87; 95% CI: 0.79–0.96; *p* = 0.005), pTNM stages 3 or 4 (sHR: 8.29; 95% CI: 2.71–25.32; *p* < 0.001) and postoperative sepsis (sHR: 6.54; 95% CI: 1.70–25.13; *p* = 0.005); see Figure 2. Figure 4 shows the 1-year adjusted cumulative incidence of cancer recurrence according to postoperative sepsis (*p* = 0.005). Finally, a Sankey diagram illustrating the relationship between different clinical features and the postoperative sepsis group suggested that patients without postoperative complications as defined by the Clavien–Dindo classification rarely experienced cancer recurrence within a year; see Figure 5.

## 5. Discussion

We report here 118 consecutive patients who underwent THE for adenocarcinoma of the EGJ over a 10-year period. Their incidence of postoperative sepsis was 24.6%, their one-year mortality was 11% and their one-year cancer recurrence was 23.7%. The factors independently associated with one-year mortality included postoperative sepsis, intraoperative driving pressure, cancer recurrence and mediastinitis, while the number of nodes removed was associated with better survival. Bacterial pneumonia, a cervical abscess and SOFA score on day 1 were independently associated with postoperative sepsis, while the intraoperative use of low-dose norepinephrine strongly protected against this event. Finally, postoperative sepsis and pTNM stages III or IV were independently associated with cancer recurrence at one year, while the number of lymph nodes removed was protective against this event.

### 5.1. Impact of Postoperative Sepsis on Long-Term Outcomes

Our study showed that postoperative sepsis was independently associated with increased one-year mortality and cancer recurrence. Although THE generally results in fewer complications than TTE [5,6], it is still associated with severe inflammatory complications, probably due to contaminations from esophageal stripping. Many of these complications are septic in nature and include conditions such as mediastinitis, severe pneumonitis and ARDS [34]. These represent a significant risk to patients’ survival and may also be indicative of underlying immune dysregulation, which can influence cancer progression [35]. Research indicates that sepsis survivors present distinct pathological phenotypes—hyperinflammatory and immunosuppressive—compared to normal phenotypes, correlating with higher mortality and re-admission rates [35]. Furthermore, evidence from a Swedish national registry suggests a link between sepsis and cancer development [36]. While an association between postoperative complications and one-year outcomes has been documented in various cancer patients [23,37,38,39], the specific context of esophageal cancer recurrence has received limited attention and remains controversial [14,17,18,40]. Two mechanisms deserve consideration: First, immune dysfunctions associated with severe septic complications may resemble those associated with cancer, creating an environment conducive to tumor progression [41]. Second, significant postoperative complications may delay the onset of adjuvant chemotherapy, impairing the effective management of residual micro-metastases and contributing to disease advancement [42]. While we did not assess whether all of the patients in both groups received adjuvant therapy, it is indeed plausible that the timely initiation of chemotherapy could have contributed to the lower recurrence rate observed in the non-sepsis group at 1 year. Future studies should explore the impact of the timing and administration of adjuvant therapies on outcomes related to postoperative complications, such as sepsis. Finally, our findings indicate that, alongside sepsis, other less obvious inflammatory complications may contribute to the recurrence of cancer. Remarkably, the data reveal that a minimal number of patients who did not experience postoperative complications went on to experience cancer recurrence (Figure 5). It is essential to clinically differentiate between these subgroups of patients.

### 5.2. Risk Factors for Postoperative Sepsis

We showed that the occurrence of a postoperative cervical abscess or pneumonia was associated with a high risk of sepsis or septic shock. While postoperative pneumonia is well recognized as a high-risk situation for postoperative sepsis after THE [43], this link is poorly documented for postoperative cervical abscesses. In this situation, in about 50% of cases, sepsis is associated with intrathoracic manifestations resulting from leakage, underlining the importance of an early diagnosis and effective drainage for favorable clinical outcomes [44]. Our results indicated that 77% of the patients with cervical abscesses had poorly drained anastomotic leaks. Cervical drains were used in 89% of our patients, although their clinical efficacy remains controversial. Indeed, a single previous randomized trial involving 40 participants reported no leaks and similar morbidity between groups, concluding that drains do not significantly help to identify leaks, as they are often removed before the leak manifests [44]. In addition, the ERAS guidelines suggest reconsidering the use of perianastomotic drains in cervical anastomoses due to moderate evidence of their insufficient benefit [25]. Nevertheless, the effective management of digestive juices using cervical drains during anastomotic leakage may reduce a patient’s Clavien–Dindo stage from grade 3 or higher to grade 2, thus potentially attenuating major complications. An ongoing randomized trial evaluating the implications of placing or not placing a drain around a cervical anastomosis could provide further information on this issue [45].

It is important to note that the intraoperative use of low-dose norepinephrine was associated with a protective effect on the occurrence of postoperative sepsis. At our institution, the use of low-dose norepinephrine is part of an overall strategy to optimize intraoperative vascular filling [46]. Indeed, for the majority of high-risk carcinologic surgeries, intraoperative vascular filling is performed with a goal-directed therapy (GDT) strategy, which falls within the scope of our ERAS protocols [32]. For esophageal surgery, we do not have an ERAS protocol specifically dedicated to vascular filling, as stroke volume monitoring cannot be performed using transesophageal ultrasound or transesophageal Doppler monitoring, which are the devices we usually use in other surgeries. The ERAS guidelines for esophagectomy do not highlight any particular specificity to THE and recommend focusing on fluid balance, while GDT may be indicated for high-risk patients; however, it is not listed in the published ERAS program [47]. In this context, we used low-dose norepinephrine, as described in a recent RCT [46], combined with sparing use of intraoperative fluids and guided by pulse pressure variations as much as possible. Our results are in line with those of an RCT by Futier et al. [46] and show that in the context of THE, low-dose norepinephrine is associated with a reduction in postoperative sepsis. Our findings differ from those of a recent RCT [48] which failed to demonstrate that GDT during esophageal surgery reduces postoperative complications. However, in this trial, the surgery was only transthoracic, unlike the present study. Finally, we found that effective and early management of organ failure, from operating room admission to the first postoperative day, as evaluated using the SOFA score, was critical in reducing postoperative sepsis [23,32].

### 5.3. Risk Factors for Long-Term Outcomes Regardless of Postoperative Sepsis

We found that intraoperative driving pressure was significantly associated with 1-year mortality. While studies indicate a correlation between driving pressure and postoperative complications, especially pulmonary issues following major abdominal surgery [34], this relationship is underexplored in esophageal surgery. In our study, intraoperative driving pressure was not elevated in patients who developed subsequent ARF compared to those without ARF. However, it was higher in patients that underwent laparoscopy than in those who did not. This finding suggests that driving pressure may serve as a surrogate marker for the local dissection conditions related to the tumor process. The potential of driving pressure monitoring as a therapeutic target or an early risk marker warrants further investigation. In addition to the intraoperative ventilatory strategy, our results suggest that the surgical technique itself can significantly influence the long-term outcomes. More specifically, the number of lymph nodes resected was found to have an independent effect on the 1-year mortality and recurrence rates, corroborating the findings of Peyre et al. [49] in cases of esophageal cancer, as well as those observed in other malignancies [50]. Indeed, extensive lymphadenectomy appears to be beneficial in eliminating microscopic residual disease, which may be overlooked in the standard assessments. This practice is in line with the North American guidelines, which recommend the resection of a minimum of 15 lymph nodes [4]. In our study, the median number of lymph nodes resected was 18; however, it should be noted that 33% of the patients had fewer than 15 nodes removed. Finally, as expected, cancer recurrence was associated with increased 1-year mortality and a higher pTNM stage (T3 or T4) [51], while mediastinitis was associated with 1-year mortality [14].

This study acknowledges certain limitations. Firstly, as it was conducted at a single institution, its findings may not be generalizable to other centers that feature different patient populations, surgical practices and perioperative protocols. Secondly, the sample size was quite small, which may have led to a reduction in the statistical power. Third, our study is limited by its focus on mortality and recurrence at one year, without exploration of longer-term outcomes such as survival beyond one year or quality of life after surgery. This limitation stems from the retrospective nature of our study and the lack of long-term follow-up data. Future studies should address these aspects to provide a more comprehensive understanding of the long-term impact of the procedures studied. Fourth, due to the retrospective nature of our study, we unfortunately lack detailed information regarding potential care limitations. Nevertheless, in the context of elective surgery, the occurrence of such limitations is usually infrequent. It is likely, however, that the prognosis of patients with more advanced stages of the disease may have influenced the level of supportive therapies administered. This is an important consideration, and further research is warranted to investigate how these variables may affect the clinical outcomes in this patient population.

## 6. Conclusions

Our study advocates for a concerted clinical effort towards the early identification of postoperative sepsis, which may serve as a pivotal strategy in reducing both mortality and recurrence in cancer patients. Further research is warranted to explore the effectiveness of early diagnostic protocols and their impact on long-term patient outcomes.

## Figures and Tables

**Table 1:** Univariate analysis for 1-year mortality: characteristics of patients.

	Overall Cohort n = 118	Alive at One Year n = 105	Dead at One Year n = 13	*p*-Value
Age (years)	64 (57–70)	64 (57–70)	64 (55–70)	0.813
Male sex	101 (85.6)	88 (83.8)	13 (100.0)	0.250
BMI	24.7 (22.6–26.6)	24.5 (22.6–26.4)	25.5 (24.2–28.1)	0.219
Charlson Comorbidity Index	4.5 (4–6)	5.0 (4.0–6.0)	4.0 (4.0–5.0)	0.681
ASA score > 2	27 (22.9)	24 (22.9)	3 (20)	1
Preoperative radiotherapy	51 (44.0)	41 (39.8)	10 (76.9)	0.025
Neoadjuvant chemotherapy	104 (88.9)	91 (87.5)	13 (100.0)	0.377
Intraoperative period				
Laparoscopy	66 (55.9)	60 (57.1)	6 (46.2)	0.648
Fluid volume (L)	4.26 (3.38–5.18)	4.26 (3.4–5.17)	4.15 (3.00–5.29)	0.760
Low-dose vasopressors	86 (72.9)	76 (72.4)	10 (76.9)	0.987
Blood transfusion	3 (2.5)	3 (2.9)	0 (0)	1
Respiratory parameters				
Tidal volume/Kg—mL/Kg	6.8 (6.3–7.1)	6.8 (6.3–7.1)	6.6 (6.2–7.3)	0.674
PEEP—cmH_2_O	5.9 (4.9–6.4)	5.9 (4.9–6.5)	5.70 (5.0–6.2)	0.836
Driving pressure—cmH_2_O	8.9 (7.4–10)	8.6 (7.35–9.70)	9.9 (8.32–12.63)	0.041
Epidural or spinal analgesia	102 (86.4)	92 (87.6)	10 (76.9)	0.527
Jejunostomy—no (%)	99 (83.9)	87 (82.9)	12 (92.3)	0.635
Nasogastric tubes—no (%)	45 (38.1)	40 (38.1)	5 (38.5)	1
Retrograde nasogastric tubes	69 (58.5)	63 (60.0)	6 (46.2)	0.527
Cervical drains	106 (89.8)	95 (90.5)	11 (84.6)	0.863
Abdominal drains	117 (99.2)	104 (99)	13 (100)	1
Postoperative period				
SAPS II score	17 (12–22)	17 (12–21)	21 (13–24)	0.245
SOFA score on				
Day 0 (ICU admission)	2 (2–3)	2 (2–3)	2.00 (2–3)	0.996
Day 1	1 (1–2)	1 (1–2)	1 (1–2)	0.628
Day 3	1 (0–2)	1 (0–2)	1 (1–2)	0.099
Clavien–Dindo stage				0.001
No complications	10 (8.5)	10 (9.5)	0	
I	19 (16.1)	17 (16.2)	2 (15.4)	
II	37 (31.4)	34 (32.4)	3 (23.1)	
IIIa	2 (1.7)	2 (1.9)	0	
IIIb	11 (9.3)	11 (10.5)	0	
IVa	30 (25.4)	26 (24.8)	4 (30.7)	
IVb	9 (7.6)	5 (4.8)	4 (30.7)	
Severe complications (IIIb, IV) *	50 (42.4)	42 (40)	8 (61.5)	0.138
Postoperative fever	56 (47.5)	45 (42.9)	11 (84.6)	0.011
Sepsis-3				<0.001
No infection	57 (48.3)	54 (51.4)	3 (23.1)	<0.001
Infection	32 (27.1)	30 (28.6)	2 (15.4)	
Sepsis	21 (17.8)	18 (17.1)	3 (23.1)	
Septic shock	8 (6.8)	3 (2.9)	5 (38.5)	
Postoperative sepsis	29 (24.6)	21 (20)	8 (61.5)	0.003
Hemorrhagic shock	4 (3.4)	4 (3.8)	0 (0)	1
Other shocks—no (%)	4 (3.4)	3 (2.9)	1 (7.7)	0.377
Acute respiratory failure	34 (28.8)	28 (26.7)	7 (46.2)	0.255
Bacterial pneumonia	29 (24.6)	23 (21.9)	6 (46.2)	0.115
ARDS	7 (5.8)	3 (2.9)	4 (30.8)	0.001
Pleural effusion	60 (50)	50 (47.6)	10 (76.9)	0.089
Pericardial effusion	6 (5)	3 (2.9)	3 (23.1)	0.014
Mediastinitis	7 (5.9)	4 (3.8)	3 (23.1)	0.033
Cervical abscesses	34 (28.8)	30 (28.6)	4 (30.8)	1
Abdominal abscesses	22 (18.6)	17 (16.2)	5 (38.5)	0.117
Anastomotic leak	33 (28)	27 (25.7)	6 (46.2)	0.222
Atelectasis	55 (47)	47 (45.2)	8 (61.5)	0.413
Invasive mechanical ventilation	9 (7.6)	5 (4.8)	4 (30.8)	0.005
Non-invasive ventilation	22 (18.6)	17 (16.2)	5 (38.5)	0.117
High-flow oxygen therapy	21 (17.8)	16 (15.2)	5 (38.5)	0.093
Acute kidney injury	24 (20.3)	20 (19)	4 (30.8)	0.331
Renal replacement therapy	2 (1.7)	1 (1)	1 (7.7)	0.524
Surgical reoperation	26 (22)	20 (19)	6 (46.2)	0.062
1-year recurrence	28 (23.7)	21 (20.0)	7 (46.7)	0.05
Pathological findings				
pT 3,4 (TNM classification)	45 (38.2)	39 (37.1)	5(38.5)	1
N1 (TNM classification)	33 (27.9)	28 (26.7)	5 (38.5)	0.586
R1 (TNM classification)	3 (2.5)	2 (1.9)	1 (7.7)	0.752
Number of lymph nodes removed	18 (13.8–23)	18 (14–23)	13 (12–14)	0.011

Results are reported as medians (interquartile ranges: IQRs) or numbers (%). ASA score = American Society of Anesthesiologists score. BMI = body mass index. PEEP = positive end-expiratory pressure. SOFA = Sequential Organ Failure Assessment. ICU = intensive care unit. ARDS = acute respiratory distress syndrome. * According to the Clavien–Dindo classification.

**Table 2:** Univariate analysis for postoperative sepsis: characteristics of patients.

	No Sepsis (n = 89)	Postoperative Sepsis (n = 29)	*p* Value
Age—yr	64 (57–69)	67 (55–71)	0.659
Male sex	77 (86.5)	24 (82.8)	0.845
BMI	24.5 (22.2- 26.3)	25.4 (24.2–27.5)	0.090
Charlson Comorbidity Index	4 (4–6)	5 (4–6)	0.297
Preoperative radiotherapy	40 (45.5)	11 (39.3)	0.723
Neoadjuvant chemotherapy	82 (93.2)	22 (75.9)	0.026
Intraoperative period			
Laparoscopy—no (%)	49 (55.1)	17 (58.6)	0.904
Low-dose vasopressors	69 (77.5)	17 (58.6)	<0.001
Blood transfusion	1 (1.1)	2 (6.9)	0.300
Respiratory parameters			
Tidal volume/Kg—mL/Kg	6.8 (6.3–7.1)	6.9 (6.3, 7.2)	0.811
PEEP—cmH_2_O	5.9 [4.8–6.5)	5.9 [5.1–6.4)	0.519
Driving pressure—cmH_2_O	8.5 (7.3–9.9)	9.2 (8.3–10.2)	0.097
Epidural or spinal analgesia	78 (87.6)	24 (82.8)	0.723
Jejunostomy	77 (86.5)	22 (75.9)	0.287
Nasogastric tubes	36 (40.4)	9 (31.0)	0.492
Retrograde nasogastric tubes	52 (58.4)	17 (58.6)	1
Cervical drains	83 (93.3)	23 (79.3)	0.710
Abdominal drains	88 (98.9)	29 (100.0)	1
Postoperative period			
SAPS II score	17 (12–21)	18 (13–28)	0.115
SOFA score on			
Day 0 (ICU admission)	2(2–3)	2 (2–4)	0.272
Day 1	1 (1–1)	2 (1–3)	0.004
Day 3	1 (0–1)	2 (1–3)	<0.001
Clavien–Dindo stage			<0.001
No complications	10 (11.2)	0	
I	19 (21.3)	0	
II	37 (41.6)	0	
IIIa	2 (2.2)	0	
IIIb	10 (11.2)	1 (3.4)	
IVa	11 (12.4)	19 (65.5)	
IVb	0	9 (31.0)	
Severe complications (IIIb, IV) *	21 (23.6)	29 (100)	<0.001
Postoperative fever	32 (36.0)	24 (82.8)	<0.001
Sepsis-3			<0.001
No infection	57 (64.0)	0	
Infection	32 (36.0)	0	
Sepsis	0	21 (72.4)	
Septic shock	0	8 (27.6)	
Hemorrhagic shock	2 (2.2)	2 (6.9)	0.541
Other shocks	0	4 (13.8)	0.003
Acute respiratory failure	9 (10.1)	25 (86.2)	<0.001
Bacterial pneumonia	10 (11.2)	19 (65.5)	<0.001
ARDS	0	7 (24.1)	<0.001
Pleural effusion	35 (39.3)	25 (86.2)	<0.001
Pericardial effusion	1 (1.1)	5 (17.2)	0.003
Mediastinitis	2 (2.3)	5 (17.2)	0.013
Cervical abscesses	19 (21.8)	15 (51.7)	0.004
Abdominal abscesses	13 (14.6)	9 (31.0)	0.059
Anastomotic leak	17 (19.1)	16 (55.2)	<0.001
Atelectasis	29 (33.0)	26 (89.7)	<0.001
Invasive mechanical ventilation	0 (0.0)	9 (31.0)	<0.001
Non-invasive ventilation	6 (6.7)	16 (55.2)	<0.001
High-flow oxygen therapy	3 (3.4)	18 (62.1)	<0.001
Acute kidney injury	15 (16.9)	9 (31)	0.106
Renal replacement therapy	0 (0.0)	2 (6.9)	0.095
Surgical reoperation	12 (13.5)	14 (48.3)	<0.001
Pathological findings			
pT 3,4 (TNM classification)	36 (40.4)	8 (27.6)	0.306
N1 (TNM classification)	23 (26.1)	10 (34.5)	0.530
R1 (TNM classification)	1 (1.1)	2 (6.9)	0.300
Number of lymph nodes removed	18 (13–22)	19.0 (14–24)	0.228

Results are reported as medians (interquartile ranges: IQRs) or numbers (%). BMI = body mass index; PEEP = positive end-expiratory pressure; SOFA = Sequential Organ Failure Assessment; ICU = intensive care unit; ARDS = acute respiratory distress syndrome; * according to the Clavien–Dindo classification.

**Table 3:** Univariate analysis for 1-year cancer recurrence: characteristics of patients.

Patients’ Characteristics	Overall Cohort n = 118	sHR (95% CI)	*p*-Value
Age	64 (57–70)	0.99 (0.95–1.03)	0.57
Male sex	101 (85.6)	1.35 (0.86–2.13)	0.19
BMI	24.7 (22.6–26.6)	0.9 (0.81–0.99)	0.048
Charlson score	4.5 (4–6)	1.05 (0.86–1.29)	0.62
Hypertension	40 (33.9)	0.39 (0.15–1.02)	0.055
Chronic kidney disease	5 (4.2)	3.24 (0.97–10.75)	0.055
Intraoperative outcomes			
Laparoscopy	66 (55.9)	0.72 (0.34–1.51)	0.381
Low-dose vasopressors	86 (72.9)	1.01 (0.45–2.30)	0.975
Blood transfusion	3 (2.5)	0.47 (0–266.64)	0.488
Respiratory parameters			
Tidal volume	461 (440–490)	1 (0.99–1)	0.414
Tidal volume/Kg	6.8 (6.3–7.1)	0.73 (0.34–1.54)	0.409
PEEP	5.9 (4.9–6.4)	0.76 (0.56–1.04)	0.088
Driving pressure	8.9 (7.4–10)	0.99 (0.82–1.19)	0.885
Postoperative			
SOFA score on			
ICU admission	2 (2.3)	0.96 (0.8–1.16)	0.679
ICU—Day 1	1 (1–2)	0.95 (0.70–1.28)	0.729
ICU—Day 3	1 (0–2)	0.988 (0.76–1.28)	0.928
Postoperative fever	56 (47.5)	1.93 (0.90–4.12)	0.09
Postoperative sepsis	29 (24.6)	1.27 (0.56–2.89)	0.566
Septic shock	8 (6.8)	1.022 (0.24–4.31)	0.976
Hemorrhagic shock	4 (3.4)	1.17 (0.16–8.61)	0.878
Blood transfusion	15 (12.7)	1.1 (0.89–1.36)	0.395
Acute respiratory failure	34 (28.8)	0.95 (0.4–2.22)	0.896
Pneumonia	29 (24.6)	0.83 (0.34–2.04)	0.682
Atelectasis	55 (47)	0.88 (0.41–1.88)	0.746
ARDS	7 (5.8)	0.52 (0.07–3.84)	0.524
Intubation	9 (7.6)	0.92 (0.22–3.87)	0.907
Non-invasive ventilation	22 (18.6)	0.68 (0.24–1.97)	0.478
High-flow oxygen therapy	21 (17.8)	0.94 (0.36.2.49)	0.911
Severe complications (IIIb, IV) *	50 (42.4)	0.68 (0.32–1.48)	0.336
Uncomplicated *	10 (8.5)	0.79 (0.19–3.35)	0.755
Anastomotic fistula	33 (28)	2.43 (0.44–2.43)	0.977
Pathological findings			
pT 3,4 (TNM classification)	45 (38.2)	7.7 (3.14–19.2)	0.000
N1 (TNM classification)	33 (27.9)	2.9 (1.28–6.01)	0.005
R1 (TNM classification)	3 (2.5)	8.57 (2.49–29.56)	0.001
Nb of lymph nodes removed	18 (13.8–23)	0.95 (0.89–1.01)	0.111

Results are reported as medians (interquartile ranges: IQRs) or numbers (%). sHR = sub-distribution hazard ratio; CI = confidence interval; ARDS = acute respiratory distress syndrome; BMI = body mass index; PEEP = positive end-expiratory pressure; SOFA = Sequential Organ Failure Assessment; ICU = intensive care unit; ARDS = acute respiratory distress syndrome; * according to the Clavien–Dindo classification.

**Table 4:** Characteristics of patients presenting with postoperative sepsis.

	Postoperative Sepsis (n = 29)
Onset of sepsis from surgery (days)	3 (2–5)
Microbiological documentation	15 (51.7)
Gram-negative bacteria	11 (37.9)
Extended-spectrum beta-lactamase	1 (3.4)
Gram-positive cocci (GPC)	10 (34.5)
Gram-positive and Gram-negative bacteria	5 (17.2)
Polymicrobial sepsis	7 (24)
Initial antibiotic treatment	29 (100)
Appropriate	13 (44.8)
Inappropriate	2 (6.9)
Without microbiological documentation (empirical)	14 (48.3)
Initial treatment with β-lactams	29 (100)
with piperacillin–tazobactam	21 (72.4)
Initial combination of b-lactams + aminoglycosides	7 (24.1)
Initial combination of b-lactams + anti-GPC antibiotics	6 (20.7)

Results are reported as medians (interquartile ranges: IQRs) or numbers (%).

## Data Availability

Data cannot be shared publicly because consent for publication of raw data was not obtained from study participants. Data are available from the Internal Review Board (IRB) of Institut Paoli Calmettes (contact via S.Maick, MAICKS@ipc.unicancer.fr) for researchers who meet the criteria for access to confidential data.

## Supplementary Materials

The following supporting information can be downloaded at: https://www.mdpi.com/article/10.3390/cancers17010109/s1, File S1: Anesthesia procedure; File S2: Surgical procedure; File S3: Pathology.
