# Supplementary material for: The Effect of Postoperative Sepsis on 1-Year Mortality and Cancer Recurrence Following Transhiatal Esophagectomy for Esophageal–Gastric Junction Adenocarcinomas: A Retrospective Observational Study
Source: Cancers (Basel). 2025 Jan 1;17(1):109. doi: 10.3390/cancers17010109 (PMC11719752; doi:10.3390/cancers17010109)
Supplement: Supplementary file 1 [file cancers-17-00109-s001.zip › cancers-3375967-supplementary.pdf]

Supplementary Materials:

File S1: **Anesthesia procedure*:***

A standardized protocol for general anesthesia was implemented for all patients. This protocol involved the controlled infusion of remifentanil and propofol for induction, followed by maintenance with desflurane or sevoflurane. Orotracheal intubation was conducted post-neuromuscular blockade with cisatracurium. Intraoperative analgesia combined epidural or spinal drug administration, ketamine infusion, and remifentanil, with or without lidocaine. Dexamethasone was administered to prevent nausea and vomiting, and multimodal analgesia was provided postoperatively. Antibiotic prophylaxis adhered to established guidelines (1). Intraoperative fluid administration was restricted to essential situations (2). Stroke volume changes were monitored via pulse pressure measurements. Intraoperative vasopressor use was classified into two categories: low-dose (boluses of ephedrine or phenylephrine, or micro-diluted continuous norepinephrine at 0.010 mg/ml) aimed at maintaining mean arterial pressure above 65 mmHg(2), and standard doses (0.5 mg/ml) when necessary.

File S2: **Surgical procedure:**

The bi-sub-costal laparotomy was the reference procedure until the end of 2016, after which it was progressively replaced by laparoscopy. In all cases, an initial surgical exploration was performed to detect potential peritoneal carcinosis or hepatic metastases. In case of uncertainty, an ex-temporaneous anatomopathological analysis was requested, and if unfavorable findings were encountered, the surgery was cancelled. Subsequently, the resection of abdominal lymph nodes was performed, involving the *en bloc* removal of tissues and lymph nodes in contact with the common hepatic artery, celiac trunk, splenic artery up to the hilum of the spleen, lesser curvature of the stomach, and cardia. Opening of the oesophageal hiatus allowed, during laparotomy, access to the lower mediastinum and its dissection. Mediastinal lymphadenectomy was performed, with the *en bloc* removal of all peri-oesophageal tissues and lymph nodes down to the carina and the aortic arch. A cervicotomy was performed preserving the recurrent nerve and the thyroid, without performing cervical lymph nodes removed. For patients operated on by laparoscopy, a short

transverse laparotomy was performed to allow extraction of the operative specimen. Subsequently, gastroplasty was performed by tubulating the greater curvature of the stomach, which was then elevated into the posterior mediastinum using a nasogastric tube as a guide. A gastro-oesophageal anastomosis was then performed at the cervical level. Mediastinal drainage via a drain positioned at the oesophageal hiatus and exteriorized in the abdomen was systematically performed. However, no thoracic drainage was implemented. The placement of a cervical drain evolved over the course of the 10-year study, as did the placement of a jejunostomy tube, the insertion of a nasogastric tube via an anterograde approach, and the insertion of a nasogastric tube via a retrograde approach with exteriorization in the abdomen

File S3: Pathology

All surgical specimens were analyzed by an experienced pathologist in accordance with the 7th edition of the American Joint Commission on Cancer Staging Manual (AJCC 7) (3) until 2017, and then with the eighth edition AJCC 8 (4).

1. Martin C, Auboyer C, Boisson M, Dupont H, Gauzit R, Kitzis M, Leone M, Lepape A, Mimoz O, Montravers P, Pourriat JL, Steering committee of the French Society of Anaesthesia and Intensive Care Medicine (SFAR) responsible for the establishment of the guidelines: Antibioprophylaxis in surgery and interventional medicine (adult patients). Update 2017. *Anaesth Crit Care Pain Med* 38(5):549–562, 2019.
2. Futier E, Lefrant J-Y, Guinot P-G, Godet T, Lorne E, Cuvillon P, Bertran S, Leone M, Pastene B, Piriou V, Molliex S, Albanese J, Julia J-M, Tavernier B, Imhoff E, Bazin J-E, Constantin J-M, Pereira B, Jaber S, INPRESS Study Group: Effect of Individualized vs Standard Blood Pressure Management Strategies on Postoperative Organ Dysfunction Among High-Risk Patients Undergoing Major Surgery: A Randomized Clinical Trial. *JAMA* 318(14):1346–1357, 2017.
3. Rice TW, Blackstone EH, Rusch VW: 7th edition of the AJCC Cancer Staging Manual: esophagus and esophagogastric junction. *Ann Surg Oncol* 17(7):1721–1724, 2010.
4. Rice TW, Ishwaran H, Ferguson MK, Blackstone EH, Goldstraw P: Cancer of the Esophagus and Esophagogastric Junction: An Eighth Edition Staging Primer. *J Thorac Oncol* 12(1):36–42, 2017.
